# Pathogenicity and virulence of hepatitis A virus

**DOI:** 10.1080/21505594.2021.1910442

**Published:** 2021-04-12

**Authors:** Rosa M Pintó, Francisco-Javier Pérez-Rodríguez, Maria-Isabel Costafreda, Gemma Chavarria-Miró, Susana Guix, Enric Ribes, Albert Bosch

**Affiliations:** a Enteric Virus Laboratory, Department of Genetics, Microbiology and Statistics, School of Biology, and Institute of Nutrition and Safety, University of Barcelona, Barcelona, Spain; b Present Address: Division of Infectious Diseases, Laboratory of Virology, University of Geneva Hospitals, Geneva, Switzerland; c Enteric Virus Laboratory, Department of Cell Biology, Physiology and Immunology, University of Barcelona, Barcelona, Spain

**Keywords:** HAV, genomic composition, codon usage, CpG, IRES, naked virions, quasi-enveloped virions

## Abstract

Hepatitis A is an acute infection of the liver, which is mostly asymptomatic in children and increases the severity with age. Although in most patients the infection resolves completely, in a few of them it may follow a prolonged or relapsed course or even a fulminant form. The reason for these different outcomes is unknown, but it is generally accepted that host factors such as the immunological status, age and the occurrence of underlaying hepatic diseases are the main determinants of the severity. However, it cannot be ruled out that some virus traits may also contribute to the severe clinical outcomes. In this review, we will analyze which genetic determinants of the virus may determine virulence, in the context of a paradigmatic virus in terms of its genomic, molecular, replicative, and evolutionary features.

## Introduction

Hippocrates, ≈400 years BC, described in his treaty “*De Morbis Internis*” an illness characterized by episodes of jaundice. Similarly, in ancient China jaundice disorders were also recognized. However, the first accurate reference to epidemic jaundice was documented by Cleghorn [1] in “Epidemic Diseases of Minorca 1744 to 1749”. In the mid-1950s, two separate entities were identified “infectious” and “serum” hepatitis referring to the mode of transmission. The “infectious” type corresponds to the hepatitis transmitted through the fecal-oral route, or enteric hepatitis, and include hepatitis A and E. The “serum” hepatitis corresponds to those parenterally transmitted, and include hepatitis B, C and D.

All types of hepatitis firstly occur as acute infections, with or without symptoms. However, while hepatitis B, C and D often develop chronic infections, hepatitis E only does it occasionally, and hepatitis A never becomes chronic.

Hepatitis A is an acute infection of the liver, mostly asymptomatic or subclinical among children under 5 years, but usually proceeding with symptoms in older children and in the adulthood [2]. The infection induces a life-long immunity in both asymptomatic and symptomatic patients, giving rise to the paradox of “hepatitis A risk” or the low and high prevalence of cases in regions of high and low endemicity, respectively [3]. A high seroprevalence of anti-HAV IgGs (high endemicity) reflects high virus circulation, mostly due to infection at an early age, and thus correlates with a low proportion of susceptible adults. On the contrary, a low seroprevalence correlates with a high vulnerability to infection in the population. One of the most recent estimations of the World Health Organization on the global burden of hepatitis A was on the 2010 foodborne cases, and reported a median number in the order of 14 million, with almost 30,000 deaths [4]. Although this is a high estimation, likely due to the method used, reflects the public health impact of hepatitis A.

A clinical case is defined by elevated serum bilirubin and aminotransferases levels that may be preceded by moderate symptoms including fever, malaise, anorexia, nausea, abdominal discomfort, dark urine and jaundice [5]. The incubation period ranges from 14 to 50 days, and clinical illness usually does not last longer than 2 months resolving completely in >99% of the cases. However, prolonged or relapsing symptoms and acute liver failure may occur in 3%–20% and in 0.015–0.15% of patients, respectively [3]. Accordingly, five distinct infection types are recognized: i) asymptomatic (mostly in children); ii) symptomatic; iii) relapsing; iv) cholestatic hepatitis, and v) fulminant hepatitis (mainly among patients with underlying chronic liver diseases).

The underlying reasons associated to these different outcomes are still not fully understood and are likely related to host factors such as the underlying hepatic diseases and age [6,7], and/or to and excessive host immune response [8]. However, virus traits potentially modulating its virulence have been described and will be the focus of this revision.

## The hepatitis A virus: A special picornavirus

The etiological agent of the human hepatitis A is the hepatitis A virus (HAV). Taxonomically, is named *Hepatovirus A* and belongs to the *Hepatovirus* genus within the *Picornaviridae* family (https://talk.ictvonline.org/ictv-reports/ictv_9th_report/positive-sense-rna-viruses-2011/w/posrna_viruses/227/picornavirales). This is a recently updated classification responding to the need of include the newly described hepatitis A viruses [9–12]. The HAV genome is a single stranded, positive-sense RNA of around 7.5 kb in length, with a long 5ʹ noncoding region (5ʹNCR) covalently linked at its 5ʹ terminus to the VPg protein (encoded in the 3B gene), which acts as a primer in the RNA synthesis during the replication [13]. The 5ʹNCR contains the internal ribosome entry site (IRES), a highly structured RNA region responsible for the recruitment of the ribosome and translation factors required for the cap-independent initiation of translation [14] of the single open reading frame (ORF) of the genome. This ORF translates as a single large polyprotein which is processed by the virally encoded protease into the structural proteins, (VP0, VP3 and VP1-pX), and the non-structural proteins (2B, 2 C, 3A, 3B, the protease 3C, the RNA-dependent RNA polymerase (RdRp) 3D, and all their precursors and intermediates) [15]. Further cleavages, independent of the viral protease, are required for the maturation of the icosahedral capsid: the VP0 is processed rendering the VP4 and VP2 proteins after RNA encapsidation, and the VP1-pX (or VP1-2A protein), is processed late in the viral lifecycle rendering the VP1 protein [16]. Near the 5´ end of the RdRp coding sequence there is a cis-acting replication element (cre) which acts as a template during the initiation of the RNA positive strands [17]. Finally, a short 3ʹNCR segment ending with a poly(A) tail is located downstream of the ORF.

**Figure 1.**
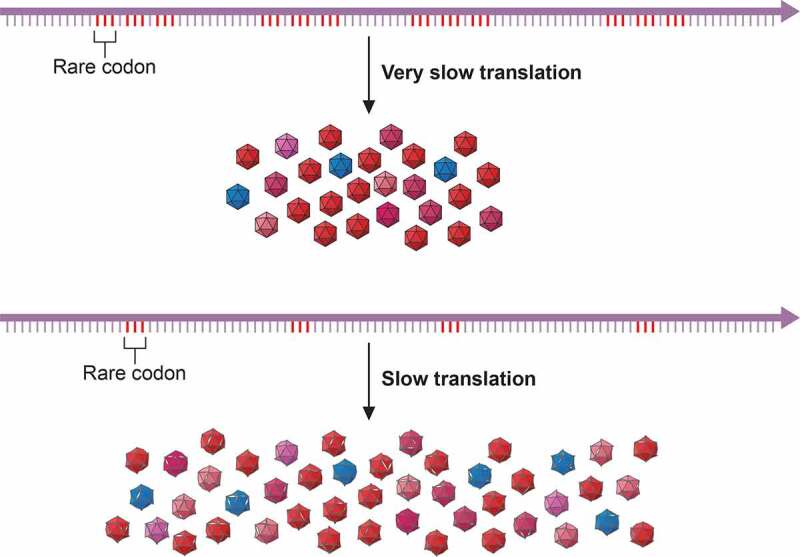
**Codon usage-driven capsid folding**. The occurrence of clusters of rare codons induce ribosome stallings which slowdown the translation speed. The codon composition of the HAV capsid coding region ensures a fine-tuned translation which results in a low production of highly cohesive capsids. Changes in codon composition increasing the rate of translation result in higher production of otherwise less cohesive capsids. HAV represents an example of the codon usage code for protein structure. Modified from [32]

However, despite this genome organization characteristic of picornaviruses, some traits contribute to the uniqueness of HAV. First, the HAV IRES is distinct among picornaviruses and constitutes the type III model [14,18,19], which is highly inefficient in directing translation [20]. Second, the HAV cre structure differs from other picornaviral elements by its relatively large size and the length of its top loop [17]. Third, while HAV encodes only for the 3C protease other picornaviruses code for additional proteases, such as the 2A protease in enteroviruses and the L protease in aphtoviruses, which not only participate in the processing of the viral polyprotein but also in the cleavage of the cellular eIF4G factor required for the cap-dependent initiation of translation [21]. Instead, the 2A protein in HAV is fused with the VP1 protein (VP1-pX) and functions in virion assembly [22], Since picornaviruses translation is IRES-dependent, the inhibition of cap-dependent translation results in the availability of the cellular translation machinery and resources almost exclusively to produce viral proteins, what is known as cellular protein shut-off. Interestingly, HAV requires an intact eIF4G factor for the initiation of translation [23,24], which supports the lack of a protease able to cleave it and in turn explains its inability to induce the cellular shut-off. This inability entails an unfair competition for the cellular translational machinery and tRNAs [25]. Consequently, to ameliorate the tRNA competition, HAV has evolved a deviated codon usage with respect to its host: highly abundant codons in the cellular genome are scarce in the virus, intermediately abundant codons in the host are abundant in the HAV genome and rare codons in the host are also rare in the virus. This distinctive codon composition of HAV, not shown in other picornaviruses, plays a crucial role in regulating the translation kinetics of the capsid coding region and in turn in controlling the folding of an outstanding resistant capsid (Figure 1) [26–28]. HAV is a paradigmatic illustration of what has been defined as the codon usage “code” for protein structure [25,29].

Another distinctive point is the occurrence of a single serotype due to severe capsid structural constraints, which prevent the emergence of new serotypes. The effect of codon usage on capsid folding may contribute to these structural constraints and in turn to the antigenic stability [25,30].

The very special genome composition of HAV is also revealed in its very low GC and GC3 contents, 37% and 26%, respectively, vs the theoretical 50% [25,31]. Additionally, HAV shows an exceptionally low dinucleotide CpG frequency (0.63% vs the theoretical 6.25%), which is not due to the low GC content since the frequency of the GpC dinucleotide (4.70%) is clearly higher and in the range of other picornaviruses [31–33]. Instead, it may result from the need to elude cellular antiviral responses. The cytosine in the CpG dinucleotides is the primary target of cellular DNA methylation, and methylated cytosines are prone to deamination generating thymines. As a result of this process over the evolutionary history of mammals, most of the remaining CpGs outside active gene promoters of somatic cells are methylated, while in the promoters are bound by CpG-binding proteins and components of the transcriptional machinery [34]. Because of the scarcity of freely accessible non-methylated CpG in the cellular DNA, sequences rich in unmethylated CpG present in a virus genome are recognized as foreign. The Toll-like receptor family (TLRs) recognizes pathogen associated molecular patterns (PAMPs), including the CpG dinucleotides, leading to a wide range of innate defense responses. While the CpG-mediated innate immune response is well established for DNA pathogens, is mostly unknown for RNA viruses [35–37]. However, no matter what the mechanism is, the low CpG content in the HAV genome may be a mechanism to escape cellular antiviral responses.

## The hepatitis A virus life cycle: Atypical virus–host interactions

**Figure 2.**
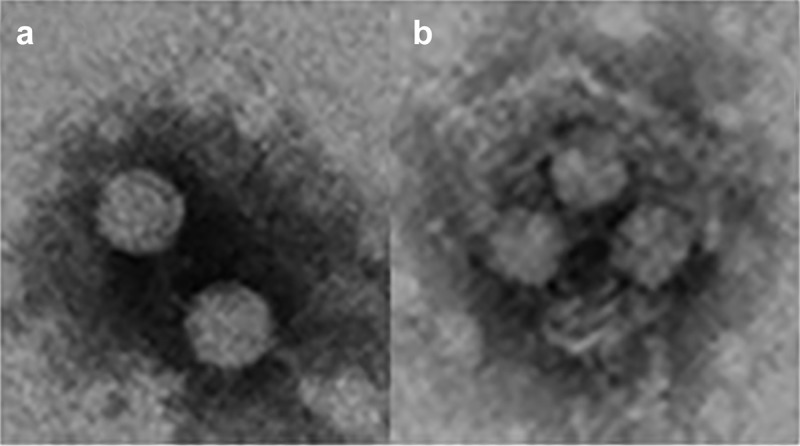
**HAV exists in a dual phenotype**. (a) Naked virions are shed in the feces of infected patients and are responsible for the fecal host-to-host transmission (b). Quasi-enveloped virions are present in the blood and are responsible for the cell-to cell transmission and occasionally parenteral host-to-host transmission. These images were obtained in our lab from supernatants of HuH7 cells infected with the HM175-43 c strain of HAV

HAV exists in a dual phenotype (Figure 2), naked and quasi-enveloped virions [38]. The quasi-enveloped virions are naked particles contained in exosome-like vesicles [39], and are the virions found in blood [40]. In contrast, naked virions are released from exosomes by the action of bile salts in the passage from the bile ducts to the gut, and are shed in feces [40,41]. Particles in the exosomes are immature and contain the VP1-pX protein; instead, naked virions in feces are mature and contain the fully processed VP1 protein [38]. However, how, and where this final processing occurs is still unknow.

The hepatitis A infection is transmitted through the fecal-oral route, and the infection cycle starts with the ingestion of naked particles. These particles are highly stable in the environment, for instance high infectious titers can still be detected after 60 days of desiccation on surfaces at room temperature [42] or in soils and water after several weeks [43], and in the harsh conditions during the transit through the stomach to the gut [27,44].

HAV infects hepatocytes, but how the virus reaches the liver from the gut is still not understood. Two hypothesis have been proposed to explain how HAV crosses the intestinal barrier into the blood: replication in epithelial intestinal cells or transcytosis through “M” cells [3,45,46]. Regarding this latter possibility, it has been proposed that naked virions may use an IgA-mediated reverse transcytosis via the polymeric immunoglobulin receptor [47]. Although the nature of the virions egressing through the basolateral membrane of these intestinal cells is unknown, they may likely be naked particles covered with IgA. Accordingly, it has been shown that HAV may infect hepatocytes via the asialoglycoprotein receptor, which binds and internalizes IgA molecules [48]. The 3D structure of mature naked HAV particles has been resolved, revealing a smooth surface lacking the usual receptor binding depression present in other picornaviruses [44], which would be in agreement with such an entry pathway. However, an open question remains unanswered in this model: the origin of anti-HAV specific IgAs in an immunologically naïve patient.

Moreover, naked particles devoid of IgAs, enter the hepatocyte by clathrin- and dynamin-dependent endocytosis in a process facilitated by integrin β1, and traffic to late endosomes where the process of uncoating is initiated [49]. Egress from hepatocytes is mostly in the form of quasi-enveloped virions [50] and through both the apical and the basolateral membranes [41]. The biogenesis of the quasi-enveloped virions involves the interaction of capsids with some endosomal-sorting complexes required for transport (ESCRT) proteins. Specifically, the interaction of two late domain motifs in the VP2 protein with the ESCRT-associated protein ALIX promotes the inward-budding of capsid-containing exosomes [38]. While these late domains are buried in the mature capsid, their accessibility may be improved in the immature capsid containing the VP1-pX protein. Actually, the VP1-pX protein also interacts with ALIX and may likewise play a role in the quasi-enveloped virion formation, despite it does not contain a late domain motif [51]. Yet, the interactions of either the VP2 late motifs or the VP1-pX protein with ALIX do not need to be mutually exclusive, and instead be part of a multi-interactive process.

The quasi-enveloped virions are also similarly endocytosed, but instead, they traffic to lysosomes where the envelope is degraded [49]. It has been recently proposed that gangliosides are essential receptors, acting on the late endosomes and lysosomes, for the release of the HAV capsids into the cytoplasm, where uncoating would occur by a still unknown mechanism [52].

A third cell-to-cell transmission pathway of HAV has been recently described, based on the delivery of capsid-free RNA genomes which are an abundant cargo of the quasi-enveloped virions [53]. The quasi-enveloped virions, which are exosomes in nature, carry phosphatidylserine molecules in their membranes, which interact with the extracellular domain of the phosphatidylserine receptor (HAVCR1), previously described as a HAV receptor [54,55], mediating their uptake by clathrin-mediated endocytosis [56]. The capsid-free RNA cargo is delivered into the cytoplasm by a mechanism of fusion between the exosome and the late endosome membranes, mediated by the interaction with the intraluminal domain of the cholesterol transporter (NPC1) located at the late endosome membrane.

Failures at any step of the infectious cycle may lead to an abortive cycle and those happening at the very beginning, such as the entry and uncoating, are particularly deleterious. Accordingly, having diverse entry pathways may be of great advantage to ensure the replication and transmission, particularly for HAV with a very slow translation.

Another strategy of HAV to warrant a successful replication is its ability to avoid the antiviral responses. The low CpG content may contribute to avoid the induction of antiviral responses, and it has been described that HAV elicits a very limited type I IFN response [57]. This response is mainly mediated by the uptake of quasi-enveloped virions into plasmacytoid dendritic cells, facilitated by the phosphatidylserine receptor, HAVCR1, present on their surface, but surprisingly does not require virus replication [58]. The cleavage of the proteins MAVS, TRIF and NEMO, which are involved in the IFN synthesis, by the HAV nonstructural proteins, 3ABC, 3CD, and 3C, respectively, explain the low IFN response [59–62]. As a result, HAV produces a very stealthy infection of the liver, leading to a new paradigm of virus–host interactions [57]. The HAVCR1 is also constitutively expressed on the surface of Treg cells, and its interaction with HAV temporally inhibits their function [63]. The produced immune imbalance permits the viral expansion with limited hepatocellular damage, a characteristic of HAV pathogenesis. The final resolution of the liver infection is mostly mediated by strong and sustained CD4 + T cells response [64].

HAV is also able to avoid the mechanisms of clearance in the blood. Some glycoproteins on the erythrocyte surface function as decoy receptors, binding pathogens and avoiding them to reach their target tissues [65]. Naked virions are able to bind to erythrocytes through the sialylglycoprotein glycophorin A, yet this binding is highly dependent on subtle conformational changes [66]. For instance, binding only occurs with the conformation acquired in acid conditions, and in consequence naked virions, if present in blood particularly at the beginning of the infection, would not be cleared by this mechanism [67]. Similarly, late in the infection, the quasi-enveloped virions are a means of protection from neutralizing antibodies in the blood facilitating the spread of the virus.

## Virulence in the context of the HAV singularity

Virulence may be defined as the damage caused by a pathogen infection, including host morbidity and mortality, i.e. the capacity to cause disease. Virulence is multifactorial and results from the complex interactions between pathogen, host and environment.

The “virulence–transmission trade-off”’ hypothesis states that virulence is an unavoidable consequence of parasite transmission [68–71]. Hence, virulence may be considered, at least partially, as a direct effect of virus replication: the higher the replication the higher the transmissibility and the higher the replication the greater the damage to the host. However, this model is considered simplistic since fitness depends not only in the replication level but also on the transmission from host-to-host, which requires adaptation for dissemination and survival between hosts [72]. Additionally, virulence may also result from immune-mediated injuries [73–75], from the infection of tissues with no relation to transmission potential [76,77], or from the existence of virulence factors particularly in viruses with large genomes [78,79]. Summarizing, virus virulence may be determined by the invasiveness, tropism, replication, modification of the host defense mechanisms, cell killing and spreading capacities.

HAV is fecal-orally transmitted and the stability of the virus outside the host is critical for the host-to-host transmission. Coupling of a deviated codon usage with an inefficient IRES results in a very slow and finely tuned translation rate, which in turn determines the protein folding for a highly robust capsid, at the cost of a low production [25,27,28]. This low production may contribute to a phenotype of moderate virulence. In addition to their antiviral effects, interferons have been linked to inflammatory diseases and immunopathologies [80,81]. In consequence, the limited interferon response of HAV may be related to the moderate clinical outcome of most hepatitis A cases.

**Figure 3.**
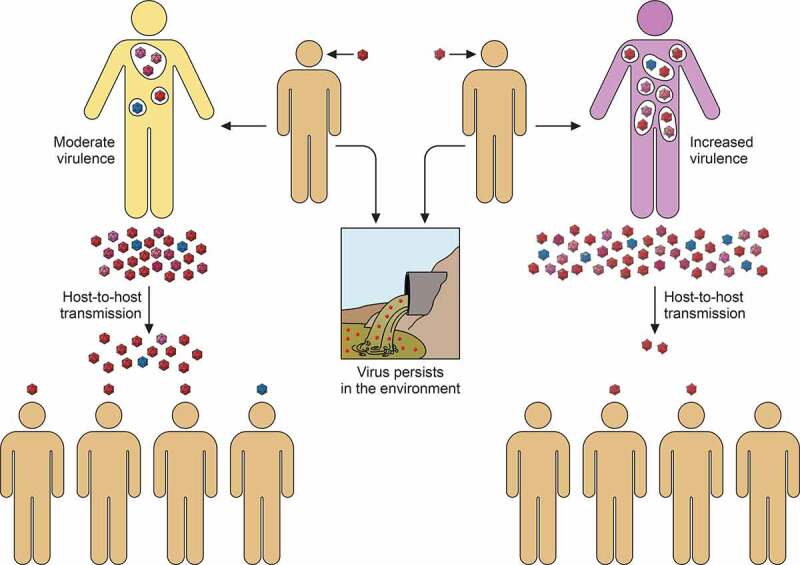
**Virulence-transmission trade-off in the context of the HAV singularity**. Left cartoon. HAV is transmitted through the fecal-oral route. During the host-to-host transmission, the virions shed in feces may persist for long periods in the environment. The high stability of the naked particles is achieved by the folding acquired through the codon usage-driven slow translation, which in combination with a very inefficient IRES results in low replication. The low virus production and the limited IFN response ends up in a moderate virulence (yellow body). Right cartoon. Changes inducing a faster replication, for instance through epistatic mutations increasing the IRES activity and optimizing the codon composition, may alter the silent dynamics of the HAV cycle increasing its virulence (purple body), and decreasing its host-to-host transmission despite a higher virus production

Overall, it can be postulated that HAV is not particularly virulent because of a strategy to safeguard its host-to-host transmission that is not strictly based on a high replication rate, combined with a very limited induction of cellular antiviral responses. However, changes in this silent dynamics might modify the phenotype of HAV virulence. Mutations resulting in an increase of replication and virus progeny, even at the expense of a less resistant and transmissible capsid, could increase virulence (Figure 3).

## Which mechanisms may explain the virulence associated to the different genotypes?

Despite HAV exists as a single serotype, human strains are distributed into three genotypes (I, II and III) and seven subgenotypes (IA, IB, IC, IIA, IIB, IIIA and IIIB) [82], although subgenotypes IA, IB and IIIA are responsible for the vast majority of infections. This classification is based on the divergence of the nucleotide sequence of the VP1-2A coding region [82,83]. Phylogenetic analyses using the sequences from this region are the most common tool used for outbreak investigations, allowing not only the identification of genotypes but also the determination of the geographic origin and the relatedness of strains involved in international outbreaks [84,85]

There are a few studies suggesting the association of some subgenotypes to fulminant hepatitis A cases. The worldwide prevalence of subgenotypes IA, IB and IIIA is of 66%, 14% and 21%, respectively, while the association of these subgenotypes to fulminant cases is of 30%, 30% and 41%, respectively [86]. These data would indicate that fulminant hepatitis is more often associated with infections of subgenotypes IB and IIIA. Particularly, subgenotype IIIA has been reported to produce more severe infections, with higher alteration of clinical parameters and requiring longer hospitalization [87]. In addition, subgenotype IIIA has been unexpectedly associated with cases in toddlers younger than 4 year-old [84]. However, the mechanism for the increased virulence of these subgenotypes is unknown. The VP1-2A coding region is highly variable, allowing the phylogenetic classification of the subgenotypes. Nevertheless, the differences in virulence of the strains belonging to each of these subgenotypes could well be related to their overall genomic composition or to other genomic regions.

The Relative Codon Deoptimization Index (RCDI) is a measure of the deviation of the codon usage of a virus with respect its host [28,88]. The RCDI of capsid sequences representative of the most common subgenotypes reveal significant differences. Subgenotypes IB and IIIA show the most and least deviated codon usage with respect the host, respectively (Table 1). The CpG content of the complete genome is significantly higher (p < 0.002) in subgenotype IIIA than in subgenotype IB, while no significant differences exist between subgenotypes IA and IB and IA and IIIA (Table 1). Yet, the ratio between the RCDI and the CpG content is significantly different between all subgenotypes. While subgenotype IB strains show the least optimized codon usage and the lower CpG content, subgenotype IIIA strains show the highest ratio, with a more optimized codon usage and a higher CpG content. In this latter case, a more efficient translation and a stronger cellular antiviral response, i.e. type I interferon, could be envisaged. However, the impairment of interferon production in the very young [89] and in the eldest [90], may render a virulent phenotype associated to a potentially higher replication.

**Table 1.** Genomic parameters of strains belonging to the most common subgenotipes

Subgenotypes^1^	RCDI^2^	CpG content^3^	RCDI/CpG content^4^
**IA**	1,6433 ± 0.013	0.59 ± 0.04	2.81 ± 0.19
**IB**	1,6839 ± 0.029	0.56 ± 0.03	3.04 ± 0.17
**IIIA**	1,5965 ± 0.006	0.63 ± 0.05	2.56 ± 0.20

^1^GenBank Accession numbers of the strains analyzed. IA: AB020565, AB020564, EU849135, EU849136, AB020567, AB020566, AB020568, AF357222, X75215, LC049341. IB: M14707, AF268396, M20273, AF314208, KX228694, KX523680, KF569906, EF406358, LC128713, HQ246217. IIIA: AB279733, AJ299464, EU849137, AB279732, EU011791, AB279734, FJ360735, AY644337, DQ991029, JQ655151.

^2^RCDI: Relative deoptimization Index. When there is a perfect match between the codon usage of a virus and its host, the RCDI value is 1. The higher the RCDI value the higher the codon usage deoptimization of the virus with respect its host. All values are significantly different by the ANOVA test (p < 0.001).

^3^ CpG content is measured as the percent of the CpG dinucleotide with respect the total number of dinucleotides in the HAV genome. Randomly, each dinucleotide should be present in a proportion of 6.25%. The CpG content of the HAV genome is very low, and the content between subgenotypes IB and IIIA is significantly different by the ANOVA test (p < 0.002).

^4^The ratio between the RCDI and the CpG content is significantly different between all subgenotypes by the ANOVA test (p < 0.001).

Nevertheless, although these findings deserve attention it should be emphasized that they are very speculative, and conclusions should be read with precaution.

## The Internal Ribosome Entry Site (IRES) as a virulence factor

In the clinical context, a pure “virulence” factor might be better considered to be one that enhances disease without influencing virus replication. Virus virulence can be measured based on illness, pathological lesions or mortality they induce. Study of the actual virulence and the mechanisms by which infection leads to disease, i.e. pathogenesis, should be studied in animal models. For HAV, these animals include tamarins [91], chimpanzees [92] and a recently developed mouse model [59].

**Figure 4.**
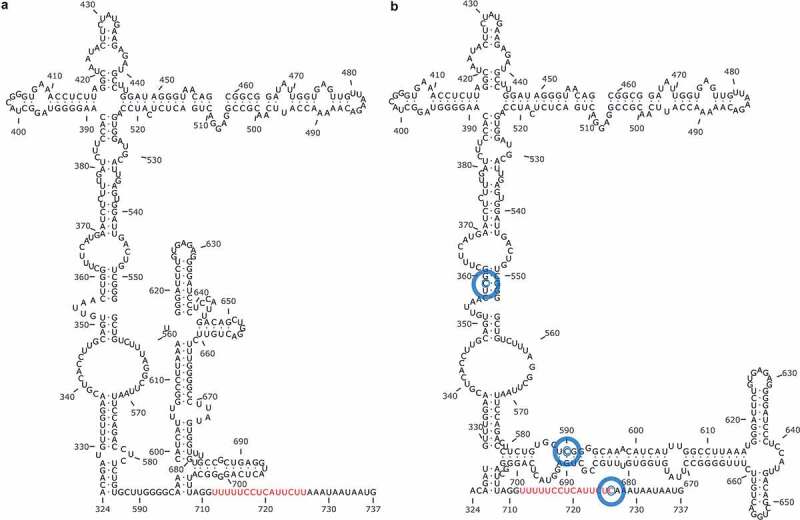
**Predicted secondary structures of the HAV IRES**. Three mutations (blue circles) induce significant structure differences in the IRES from a slow-growing strain (a) and a fast-growing strain (b). The second polypyrimidine tract which precedes the AUG is shown in red. Extracted from [93]

In HAV, the inefficient IRES and the deviated codon usage separately, and particularly in combination, are responsible for a very slow translation rate. The occurrence of three mutations in the IRES (U359C, U590C and U726C in the HM175 strain), which induce a change of its secondary structure (Figure 4), significantly increase its activity, particularly when they are combined with changes of codon composition in the capsid coding region [28,93]. Remarkably, a strain bearing all these changes shows a fast-growing phenotype [93]. Although not tested in an animal model yet, this strain would potentially be more virulent. For instance, for poliovirus it has been proposed that the mutation C472U in the IRES stem-loop V [94] as well as interactions between the stem-loops V and VI, are major determinants of its neurovirulence [95]. Additionally, experimental evolution studies of the oral poliovirus vaccine serotype 2 showed the occurrence of three “gate-keeper” mutations, preceding all other substitutions, associated with an increase of virulence in mice [96]. Two of these “gate-keeper” mutations (U398C and A481G) are located in the poliovirus IRES domains IV and V. Similarly, the mutations increasing the HAV IRES activity are also located in the homologous domains IV, V and VI [93,96,97]. Nevertheless, it has been proposed that poliovirus neurovirulence is not related with the efficiency of the IRES-driven initiation of translation, but rather in requirements for sequences contained within the IRES for the viral RNA replication [98]. No data exists on such requirements for HAV. Consequently, it is difficult to define whether the HAV IRES is a true virulence factor or merely modulates virulence through the initiation of translation and in turn in virus replication.

This fast-growing strain of HAV was present at a very low frequency in the mutant swarm of a population long adapted to grow in conditions of moderate transcription shutoff, and was rescued only after a process of competition with a population adapted to grow in conditions of high transcription shutoff [93]. Additionally, this fast-growing population acquired some codon replacements in the capsid coding region, optimizing their frequency with respect the cell codon usage, which showed an epistatic effect toward the fast replication [93]. Fortunately, such a process is not anticipated to happen in natural infections, since it would require patients with unexpectedly long infections and under treatments with drugs inducing the cell shutoff such as the actynomicin D to be able to select such a combination of mutations.

Few clinical studies have addressed the role of mutations located in the IRES region in the outcome of the hepatitis A infection. A study with twelve patients with severe and benign hepatitis could not identify mutations in the IRES specifically associated with either form of the infection [99]. In contrast, another study comparing the sequences from eighty-four patients with acute benign, acute severe and fulminant hepatitis, found higher nucleotide variation (between nucleotides 200 and 500) among the benign form [100]. This constraint in the nucleotide variability in viruses from severe and fulminant hepatitis suggests that IRES variability may act as an attenuation factor.

Despite the underlying mechanism would still be unknown, an independent study supported this observation. A deep-sequencing analysis of the 5ʹNCR of viruses isolated from five patients from an outbreak in the men-having-sex-with-men group [85] revealed an association between IRES variability and the severity of the infection (Table 2). Although none of these patients developed a fulminant hepatitis, they showed differences in severity. There was a significant positive linear correlation (Pearson and Spearman correlation) between viremia and alanine transferase levels, and a significant non-linear negative correlation (Spearman correlation) between alanine transferase levels and IRES variability, referred as the normalized ratio between the number of haplotypes and the genome copy numbers in blood. Even though it is difficult to know what comes first, it is reasonable to think that lower IRES variability may result in a more efficient translation, likely by purifying selection of genomes with non-optimal IRESs, which in turn would result in higher viremia and consequently in more severity. More widespread studies are required to confirm this association and its clinical relevance.

**Table 2.** **Clinical parameters, viral load and IRES variability in a cohort of five hepatitis A patients from an outbreak in the men-having-sex-with-men**. These patients were not vaccinated and HIV-non-infected

Patient	ALT^a^ (U/L)	Bilirubin^a^ (mg/dL)	Illness duration^a^ (days)	Genomes^a^ (copies/mL)	Number of haplotypes^b^ (per 10^5^ genome copies)
**M30**	528	5.9	14	1.2 x 10^5^	2.6
**M2**	1813	4.0	22	3.6 x 10^5^	0.68
**M47**	3148	6.5	28	3.3 x 10^6^	0.20
**M9**	4804	6.8	28	1.1 x 10^7^	0.016
**M10**	9000	8.9	34	1.3 x 10^8^	0.016

^a^These results were taken from [85].

^b^These results were obtained in the same study reported in [85] but have been analyzed for the first time in this review.

## May the biogenesis of the quasi-enveloped virions be considered a virulence factor?

It has been suggested that the VP1-2A and 2C genes, separately and particularly in combination, are virulence factors, as mutations in these genes attenuate the phenotype of a wild-type strain in tamarins [91]. Yet, the “attenuating” mutations described, dramatically reduced the replication capacity of the virus, leading to a 1000-fold reduction of virus fecal shedding along with less disease. Thus, these mutations appear to restrict virus replication, rather than alter the virus-host interactions leading to liver damage. However, a tempting speculation arises in relation to the proposed VP1-pX involvement in the biogenesis of the pseudo-enveloped virions [51]: could mutations in this region influence virus release and hence virulence?

Additionally, when compared to subgenotypes IA, subgenotype IIIA and to a lesser extent subgenotype IB strains show more amino acid replacements in the VP1-2A region, which could be related to their higher virulence. Presently, there are no experimental data in animal models confirming such a possibility, which may be partially explained by the difficulties to grow wild-type viruses in cell cultures, and thus the unavailability of virus inocula of such strains.

The actual function of 2C protein, and thus its relationship to virulence, is not well characterized, but sequences of strains from fulminant and severe hepatitis cases show fewer amino acid substitutions than the sequences from acute hepatitis cases [101]. This constraint might suggest an association between the severity of hepatitis A and the amino acid composition of 2C, although specific residues linked to the severity are yet to be identified. Again, data on animal models is lacking.

## Conclusion

Virulence is determined by the invasiveness, tropism, replication, modification of the host defense mechanisms, cell killing and spreading capacities of a pathogen. The host-to-host transmission of HAV is ensured by an outstandingly stable capsid whose solid folding is accomplished by a finely-tuned slow translation controlled by a special codon composition and an inefficient IRES. The consequence is a slow virus replication. Additionally, the HAV infection induces limited antiviral innate cell responses. The combination of a slow replication and a limited antiviral response results in low virulence.

However, a small proportion of severe and fulminant cases do occur. The reason of these serious clinical outcomes is probably multifactorial being the result of host and virus factors. The IRES activity may presumably act as a virulence factor, and it is tempting to speculate that the ability to interact with the biogenesis of the pseudo-enveloped particles may also be a determinant of pathogenesis. Infection of patients with impaired innate antiviral responses with strains bearing mutations affecting the mentioned virulence factors may result in severe hepatitis A cases.
